# Overexpression of CD151 Predicts Prognosis in Patients with Resected Gastric Cancer

**DOI:** 10.1371/journal.pone.0058990

**Published:** 2013-03-22

**Authors:** Yue-Ming Yang, Zhong-Wei Zhang, Qing-Meng Liu, Yi-Feng Sun, Ji-Ren Yu, Wei-Xing Xu

**Affiliations:** 1 Department of Gastrointestinal Surgery, Shaoxing Hospital of First Affiliated Hospital of Medical School of Zhejiang University and Shaoxing Second Hospital, Shaoxing, P. R. China; 2 Department of Pathology, Shaoxing Hospital of First Affiliated Hospital of Medical School of Zhejiang University and Shaoxing Second Hospital, Shaoxing, P. R. China; 3 Department of Gastrointestinal Surgery, First Affiliated Hospital of Medical School of Zhejiang University, Hangzhou, P. R. China; INRS, Canada

## Abstract

**Purpose:**

The tetraspanin CD151 acts as a promoter of metastasis and invasion in several tumors. However, the role of CD151 in human gastric cancer (HGC) remains unclear.

**Methods:**

Twenty HGC specimens and matched nontumor samples, human gastric epithelial cells (HGEC), and four gastric cancer cell lines were used to analyze CD151 expression. Short hairpin RNA-mediated downregulation of CD151 expression in HGC cells was performed to examine the role of CD151 in the proliferation and metastasis/invasion of HGC cells *in vivo* and *in vitro*. The relationship of CD151 with integrin α3 in HGC cells was investigated by silencing integrin α3 followed by co-immunoprecipitation and immunofluorescence staining. Finally, the prognostic value of CD151 and integrin α3 was evaluated by immunohistochemistry in tissue microarrays of 76 HGC patients.

**Results:**

CD151 was expressed at higher levels in HGC tissues and HGC cells than in nontumor tissues and HGEC cells. Down-regulation of CD151 by vshRNA-CD151 impaired metastasis and invasion of HGC-27 cells, but did not affect cell proliferation. CD151 formed a complex with integrin α3 in HGC cells. CD151-cDNA transfection rescued the metastatic potential and invasiveness of HGC-27-vshCD151 cells, but not those of HGC-27-vshintegrin α3 cells *in vitro*. Clinically, CD151 overexpression was significantly correlated with high TNM stage, depth of invasion and positive lymph node involvement (*p*<0.05), and high levels of integrin α3 were associated with large tumor size, high TNM stage, depth of invasion and lymph node involvement (*p*<0.05). Importantly, the postoperative 5-year overall survival of patients with CD151^low^ and/or integrin α3^low^ was higher than that of patients with CD151^high^ and/or integrin α3^high^.

**Conclusion:**

CD151 is positively associated with the invasiveness of HGC, and CD151 or the combination of CD151 and integrin α3 is a novel marker for predicting the prognosis of HGC patients and may be potential therapeutic targets.

## Introduction

Human gastric cancer (HGC) is the most frequent cause of cancer-related death [1]. The incidence of HGC was estimated to be 934,000 cases per year with 56% of new cases occurring in East Asia, including 41% in China and 11% in Japan [2]. Although the global incidence of GC has decreased in recent years, its mortality rate in China is the highest among all tumors and represents 25% of GC mortality worldwide [3]. Despite recent advances in chemotherapy and surgical techniques, the 5-year overall survival (OS) rate in China is low at 40%. Most HGCs are diagnosed at stage III or IV, and the rate of lymph node metastasis from GC is high (50–75%) [4]. The pathogenesis of HGC is multifactorial including genetic predisposition and environmental factors. Several genetic alterations are associated with the predisposition to HGC, including those involving tumor suppressor genes, oncogenes, cell adhesion molecules, growth factors, and genetic instability [5]. Therefore, achieving a better understanding of the molecular mechanisms involved in HGC and identifying valuable diagnostic markers and novel therapeutic strategies is of great clinical significance.

Tetraspanins are cell-surface proteins that span the membrane four times, and are found in several cell types in many organisms. They display numerous properties indicative of their physiological importance in cell adhesion, motility, activation and proliferation, as well as their contribution to pathological conditions such as metastasis and pathologic angiogenesis [6], [7], [8]. CD151 is a cell surface glycoprotein belonging to the tetraspanin superfamily that was first shown to promote metastasis in a study in which an unknown antibody specifically inhibited metastasis formation in a human epidermoid carcinoma *in vivo* [*9*]. The antibody recognized CD151 and inhibited cell migration without affecting adhesion or proliferation. Small interfering RNA (siRNA) mediated downregulation of CD151 expression in primary melanocytes resulted in the loss of motility, while it had little effect on the steady-state levels of integrins. Moreover, these alterations could be reversed when CD151 was re-expressed [10]. Overexpression of CD151 was shown to activate integrin and growth factor receptor dependent signaling pathways, which resulted in the increased motility and invasiveness of cancer cells [11]. This process was also suggested to contribute to the activation of pathways mediated by small GTPases, which increases GTP binding to Cdc42 and Rac, organizers of the cell cytoskeleton. The adhesion-dependent activation of Ras, ERK/MAPK1/2 and protein kinase B (PKB)/Akt has been shown to be modulated by CD151 [7], [12]. The wide range of functions of CD151, and in particular its involvement in the invasiveness and metastasis of cancer cells, suggest that a better understanding of its expression and role in HGC may be important.

In the present study, we investigated the expression of CD151 in human gastric epithelial cells (HGEC), HGC cell lines, HGC samples, and adjacent nontumorous tissues. In addition, we explored the effect of siRNA silencing of CD151 on the proliferation, invasiveness and metastatic ability of HGC-27 cells, and examined the relationship between CD151 and integrin α3. Finally, the expression of CD151 and integrin α3 was examined by immunohistochemistry in a tissue microarray consisting of 76 cases of HGC, and the prognostic role of CD151 and/or integrin α3 in HGC was investigated.

## Materials and Methods

### Cell Lines

HGEC and HGC cell lines, including HGC-27, AGS, MKN28, and MGC803, were obtained from the American Type Culture Collection and kept in our laboratory. All cell lines were maintained in Dulbecco’s Modified Eagle’s medium (DMEM, Invitrogen) supplemented with 10% fetal bovine serum (Hyclone) at 37°C in a humidified incubator under 5% CO_2_.

### Patients and Follow-up

Twenty fresh tumor samples from areas close to the tumor margin and matched non-tumor tissues (more than 3 cm away from the tumor) were blindly obtained from consecutive patients with HGC who underwent curative resection between February 2009 and November 2010 at Shaoxing Second People’s Hospital (Shaoxing, China). Another 76 patients with gastric cancer who underwent R0 resections with extended lymph node dissection (D2) between September 2005 and September 2008 at Shaoxing Second People’s Hospital were enrolled in this study. The evaluation of resected specimens was performed in accordance with the guidelines of the Japanese Gastric Cancer Association (1998). Each standard resection involved the removal of group 1 and 2 lymph nodes (range 36−60, mean 47.6). Stage classification was performed according to the TNM classification for HGC (UICC). Specimens were selected on the basis of the availability of suitable formalin-fixed, paraffin-embedded tissues and complete clinicopathologic and follow-up data from the patients. The characteristics of the study subjects were summarized in Table 1. This study was approved by the Shaoxing Second People’s Hospital Research Ethics Committee and written informed consent was obtained from all individuals. None of the patients received chemotherapy or radiation therapy before or after surgery as part of an adjuvant program. Follow-up data collection was terminated on September 2012. The median follow-up was 43.0 months (range 6−78 months). Follow-up procedures consisted of interim history, physical examination, assessment of tumor markers (CEA, CA-199), abdominal ultrasonography and chest X-ray every 2–3 months or CT every 6 months. Overall survival (OS) was defined as the interval between surgery and death or between surgery and the last observation in surviving patients. The data were censored at the last follow-up for living patients.

**Table 1:** Correlation between CD151/Integrin α3 and clinicopathological characteristics in 76 human HGC patients.

		CD151		*P* value	Integrin α3		*P* value
		Low (38)	High (38)		Low (39)	High (37)	
Gender	Female	25	22	0.129	26	21	0.172
	Male	13	16		13	16	
Age (y)	≤65	21	22	0.427	20	23	0.369
	>65	17	16		19	14	
Tumor size(cm)	≤5.5	32	17	**0.021**	37	12	**3.46E−4** *
	>5.5	6	21		2	25	
Differentiation	I-II	25	23	0.096	23	25	0.078
	III	13	15		16	12	
Depth of invasion	T1	9	16	**0.004**	18	7	**0.001**
	T2–T4	29	22		21	30	
Lymph nodule involvement	Positive (N1/N2/N3)	14	18	**0.028**	16	16	**0.040**
	Negative (N0)	24	20		23	21	
Stage	I	21	3	**0.002** *	20	4	**0.005** *
	II/III/IV	17	35		19	33	

**Abbreviations:** Chi-square tests for all the analyses,

* Fisher’s Exact Test.

### RNA Extraction and Reverse Transcription-polymerase Chain Reaction (RT-PCR)

Total RNA was extracted using the TRI Reagent (Sigma) according to the manufacturer’s protocol. RNA was then treated with DNaseI (Roche Diagnostics), purified through an RNeasy column (Qiagen) and electrophoresed to determine the integrity of the RNA before use in 5′-RACE experiments. Complementary DNA (cDNA) was synthesized from 2 µg of total RNA using random hexamers (Proligo) and SuperScript III Reverse Transcriptase (Invitrogen). RT-PCR was carried out on a panel of cell lines and tumor samples. Primers used for PCR were as follows: CD151, 5′-ACTTCATCCTGCTCCTCATCAT-3′ and 5′-TCCGTGTTCAGCTGCTGGTA-3′; integrin α3, 5′-CGAGAGGAAAGAGGAAGTAGGGGGT-3′ and 5′-ATGAAGAAGGAGTGAGGGGTGAGCA-3′; Glyceraldehyde 3-phosphate dehydrogenase (GAPDH), 5′-GGCATCCTGGGCTACACTGA-3′ and 5′-GTGGTCGTTGAGGGCAATG-3′. The RT-PCR conditions were as follows: 10 min at 94°C, denaturation at 94°C for 20 s, annealing at 59°C for 30 s, and extension at 72°C for 60 s. The expression of CD151 and integrin α3 relative to the housekeeping gene GAPDH was analyzed by density analysis using Band Scan v5.0 software. All experiments were performed in triplicate.

### Immunoblotting and Immunofluorescence Assay

Total cell extract protein (30 µg) was separated by SDS-polyacrylamide gel electrophoresis, transferred onto polyvinylidene difluoride membranes, and incubated with the corresponding antibodies. The membranes were developed with the enhanced chemiluminescence method (Pierce, Rockford, IL, USA). Mouse anti-human CD151(11G5a, 1∶200; Serotec, UK) and anti-integrin α3 monoclonal antibodies (P1B5, 1∶300; Chemicon International, Temecula, CA) were used to detect the expression of CD151 and integrin α3, respectively. GAPDH (1∶5,000; Chemicon, USA) was used as an internal control. All experiments were performed in triplicate.

HGC-27 cells were used to detect the location of CD151 and integrin α3 as described previously [13]. Mouse anti-human CD151 monoclonal antibody (11G5a, 1∶200; Serotec, UK) and mouse anti-human integrin α3 antibody (P1B5, 1∶300; Chemicon International, Temecula, CA) were used. The slices were analyzed by fluorescence microscopy (Leica Microsystems Imaging Solutions).

### Transfection of Lentiviral Vectors with Small Hairpin RNA Against CD151 and Integrin α3

The pGMLV/Neo-shRNA-CD151 vector was constructed according to the manufacturer’s instructions (pGMLV, a small hairpin RNA (shRNA)i Vector, Shanghai Genomeditch Co. Ltd). Three shRNA-CD151 lentiviral vectors (pGMLV-GFP-shRNA -CD151) were generated to silence the expression of CD151 in HGC-27 cells (shRNA-CD151-HGC-27). The shRNA targeting sequences for CD151 were as follows: #1, 5′-CATGTGGCACCGTTTGCCT-3′; #2, 5′-TACCTGCTGTTTACCTACA-3′; #3, 5′-CATACAGGTGCTCAA TAAA-3′. The shRNA targeting sequence for integrin α3 was as follows: 5′- CCTCTATATTGGGTACACGAT-3′ **(**Shanghai Genomeditech, Shanghai, China). Stably transfected clones were characterized by RT-PCR and analyzed by immunoblotting for the expression levels of the CD151 and integrin α3 proteins.

### MTT Assay, Cell Migration, and Matrigel Invasion Assays

Cells were seeded into 96-well plates (2,000 per 200 µl-well) and incubated for 24, 48, and 72 h. A 20 µl volume of MTT solution was added at the indicated time points and incubated for 4 h. The medium (200 µl DMEM containing 10% fetal bovine serum) was then replaced by 150 µl DMSO, plates were shaken for 10 min and the absorbance at 490 nm was measured to determine the number of viable cells in each well. All experiments were performed three times.

Cell migration was evaluated using a wound-healing assay. Cells were grown to 80–90% confluence in 24-well plates. A wound was made by dragging a plastic pipette tip across the cell surface. The remaining cells were washed three times to remove cell debris and incubated at 37°C with serum-free medium. At the indicated times, migrating cells at the wound front were photographed and compared. Three separate experiments were performed.

Cell invasion assays were performed using 24-well transwells (8 µm pore size; Millipore) precoated with Matrigel (Falcon 354480; BD Biosciences). A total of 1×10^5^ cells was then suspended in 500 µl DMEM containing 1% FBS and added to the upper chamber, while 750 µl DMEM containing 10% FBS was placed in the lower chamber. After 48 h of incubation, Matrigel and cells remaining in the upper chamber were removed by cotton swabs. Cells on the lower surface of the membrane were fixed in 4% paraformaldehyde and stained with Giemsa. Cells in 5 microscopic fields (magnification, ×200) were counted and photographed. All experiments were performed in triplicate.

### In Vivo Metastasis Assays

For in vivo metastasis assays, MGC-803-Mock, MGC-803-vshRNACD151 and MGC-803-vshRNA CD151-cDNA-CD151 cells were transplanted into nude mice (5-week-old BALB/c-nu/nu, 5 per group, 1×10^6^ cells for each mouse) through the lateral tail vein [14]. After 7 weeks, mice were sacrificed. Their lungs were removed and subjected to hematoxylin and eosin (H&E) staining. All research involving animals was performed in compliance with protocols approved by the Shaoxing Second People’s Hospital Animal Care Commission.

### Co-immunoprecipitation (Co-ip) Assays

Cells were lysed with RIPA lysis buffer supplemented with 40 mM NaF, 100 µM Na_3_VO_4_, and Complete Protease Inhibitor (Roche). After removing the insoluble material by centrifugation at 12,000×g, the precleared lysates were incubated with primary mAb pre-absorbed protein A- and G-Sepharose beads (Pierce Biotechnology) overnight at 4°C. The precipitates were washed three times with lysis buffer, boiled in 2×SDS sample buffer for 5 minutes, and proteins were resolved by SDS-PAGE on 10% gradient gels. Subsequent immunoblots were probed with the appropriate antibody and detected by ECL.

### Construction of Tissue Microarrays and Immunohistochemistry

Tissue microarrays were constructed as described previously [15]. Briefly, all samples from HGC patients were reviewed histologically by hematoxylin & eosin staining, and representative areas were premarked in the paraffin blocks, away from necrotic and hemorrhagic materials. Duplicates of 1-mm-diameter cylinders from two different areas, the tumor center and the nearest noncancerous margin (designated as intratumor and peritumor, respectively; a total of four punches) were included in each case, along with different controls, to ensure reproducibility and homogenous staining of the slides (Shanghai Biochip Co. Ltd, Shanghai). Thus, four different tissue microarray blocks were constructed, each containing 140 cylinders. Sections 4 µm thick were placed on slides coated with 3-aminopropyltriethoxysilane. Monoclonal mouse anti-human CD151 (11G5a, 1∶200; Serotec, UK) and mouse anti-human integrin α3 (P1B5, 1∶300; Chemicon International, Temecula, CA) antibodies were used to detect the expression of CD151 and integrin α3. Images were captured by the Leica QWin Plus v3 software. The intensity of positive staining was measured as described. The intensity of CD151 and integrin α3 was classified into two levels of expression according to the mean area of positive staining as the cutoff value as follows: CD151^high^, >50% of the tumor section; and integrin α3^high^, >25% of the tumor section; CD151^low^, <50%, and integrin α3^low^, <25% [15].

### Statistical Analysis

Statistical analysis was performed with SPSS 16.0 software (SPSS). Values were expressed as the mean ± standard deviation. For immunohistochemical markers, the cutoff for the definition of subgroups was median intensity value. The χ^2^-test and Student’s t-test were used for comparisons between groups. OS was defined as described previously [15]. Prognostic significance was assessed using Kaplan-Meier survival estimates and log-rank tests. Cox’s proportional hazards regression model was used to analyze the independent prognostic factors. All tests were two-tailed and *p*<0.05 was considered statistically significant.

## Results

### CD151 is Overexpressed in HGC

**Figure 1:**
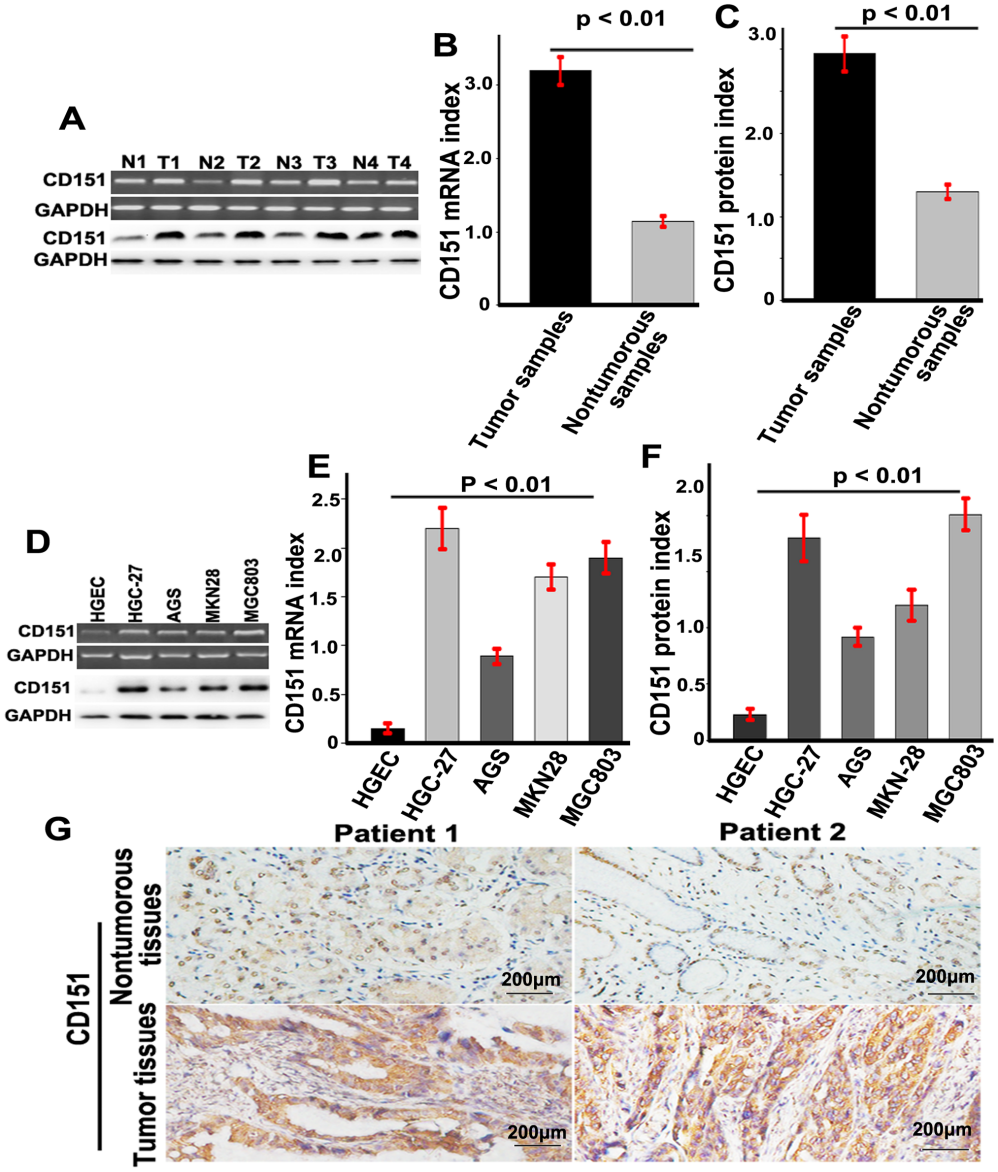
CD151 was overexpressed in HGC. (**A**) The expression of CD151 mRNA and protein in GC samples and the matched nontumorous samples; (**B and C**) a histogram showed CD151 mRNA in GC samples and the matched nontumorous samples (p<0.05); (**D**) RT-PCR and immunoblotting analyzed the expression of CD151 in HGEC and HGC-27, AGS, MKN28 and MGC803 cells (p<0.05); (**E and F**) A histogram showed CD151 mRNA and protein in HGEC and HGC-27, AGS, MKN28 and MGC803 cells (p<0.05).

CD151 expression was analyzed by RT-PCR and immunoblotting in HGC tumor and matched nontumor tissues. CD151 was expressed at low levels in nontumor tissues compared with HGC tissues. As shown in Fig. 1**A,B and C**, the relative expression of the CD151 protein in HGC samples was 2.95±0.21 (range, 1.13 − 3.59) compared to 1.30±0.09 (range, 1.00−1.81) in nontumor samples, and the difference was statistically significant (*p*<0.01). Furthermore, CD151 expression levels were significantly lower in HGEC than in HGC-27, AGS, MKN28 and MGC803 cells (p<0.05). No differences in CD151 expression levels were detected among HGC cells (**Fig. 1D, E and F**). Immunohistochemstry (IHC) analyses confirmed that the CD151 protein was expressed at higher levels in HGC tissues than in matched nontumor tissues (**Fig. 1G**).

### CD151 Promoted the Invasion and Metastasis of HGC Cells *in vitro and in vivo*

**Figure 2:**
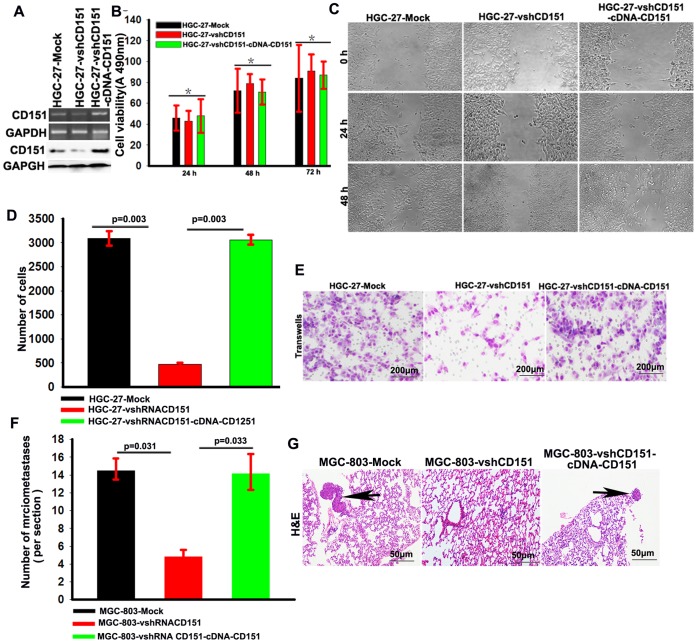
CD151 promoted the invasion and metastasis of HGC cells *in vitro and vivo.* (**A**) The expression of CD151 in HGC-27 cells by RNA interference and cDNA-CD151 transfection; (**B**) The down/or up-regulation of CD151 have no influence on cell proliferation (*p*>0.05); (**C**) The wound-healing assay revealed that an evident delay in the wound closure rate of HGC-27-vshRNA-CD151 cells was found at 24 and 48h, compared with HGC-27-Mock cells, while it was recovery by cDNA-CD151 transfection; (**D and E**) Matrigel invasion assays showed that down/or up-regulation of CD151 expression was accompanied by a descend/or ascend invasion of HGC cells in vitro; (**F** and **G**) Serial sections from mouse lung showed the metastasis ability of cancer cells expressing different CD151 (Scale bar: 50 µm).

To examine the role of CD151 in HGC cells, we modified the expression of CD151 in HGC-27 cells by RNA interference and cDNA-CD151 transfection (**Fig. 2A**). The results of the wound healing assay showed a delay in the wound closure rate of shRNA-CD151-HGC-27 cells at 48 h compared with HGC-27-Mock cells, which was recovered by cDNA-CD151 transfection (**Fig. 2B**). Down-regulation of CD151 had no significant effect on cell proliferation (*p*>0.05, **Fig. 2C**). However, the downregulation of CD151 expression impaired the invasiveness of HGC-27 cells **(** **Figs. 2D, E**).

To further explore the role of CD151 in tumor metastasis in vivo, MGC-803-Mock, MGC-803-vshRNACD151 and MGC-803-vshRNACD151-cDNA-CD151 cells were transplanted into nude mice through the lateral tail vein. Histologic analysis of the lungs of mice confirmed that the down-regulation of CD151 suppressed lung metastasis formation. The numbers and size of lung metastasis nodules were significantly decreased in the MGC-803-vshRNACD151 group when compared with MGC-803-Mock and MGC-803-vshRNACD151-cDNA-CD151 cells (**Figs. 2** **F, G**). Taken together, our results suggest that CD151 is a positive regulator of gastric cancer metastasis.

### CD151 Forms a Complex with Integrin α3 and Silencing of Integrin α3 Impairs HGC Cell Invasion Induced by CD151 Overexpression

**Figure 3:**
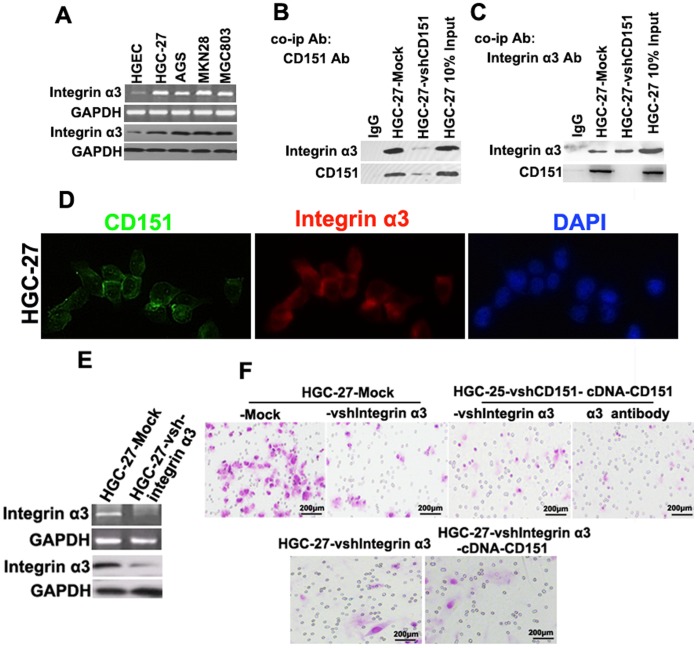
CD151 form a complex with integrin α3 and the interference of integrin α3 impair the HGC cells invasion endowed by CD151 overexpression. (**A**) The expression of integrin α3 were much higher in HGEC and HGC cells; (**B and C**) The co-IP identified that the integrin α3 form a complex with integrin α3; (**D**) Double staining indicated the CD151 and integrinα3 co-localized on the membrane of HGC-27 cells; (**E**) The down-regulation of integrin α3 in HGC cells; (**F**) Matrigel invasion assays showed that down-regulation of integrin α3 was accompanied by a descend invasion of HGC cells, the integrin α3 interference and integrin α3 antibodies inhibited the cells invasion endowed by CD151.

The involvement of tetraspanins in the integrin-mediated regulation of cancer metastasis has been demonstrated, and α3β1 integrin has been shown to promote GC invasion and metastasis [16]. Therefore, we investigated the relation between integrin α3 and CD151 in HGC cells. As shown in **Fig. 3A** **,** integrin α3 was expressed at higher levels in HGC cells than in HGEC cells. Co-immunoprecipitation experiments showed that CD151 formed a complex with integrin α3 in HGC-27 cells (**Figs. 3B, C**). Moreover, CD151 and integrin α3 co-localized on the plasma membrane of HGC-27 cells as shown by immunofluorescence (**Fig. 3D**).

To further examine the involvement of CD151 in integrin α3 function, cells were subjected to RNA interference or cDNA transfection and analyzed by Transwell experiments after replacing the matrix gel with laminin-332 [17]. The results showed that silencing of integrin α3 markedly inhibited the invasion of HGC-27 cells, and CD151 cDNA transfection rescued the invasive ability of HGC-27-vshRNACD151, but not that of HGC-27-vshRNA integrin α3 cells. Furthermore, integrin α3 antibodies inhibited the invasion of HGC-27-vshRNACD151-cDNA-CD151 cells (**Figs. 3E, F**). These results suggested the coordinated function of the CD151-integrin α3 complex, and indicated that high levels of CD151 and integrin α3 are associated with the increased metastatic potential of GC cells.

### Expression of CD151 or Integrin α3 is Positively Associated with Malignant Phenotypes of HGC by Immunohistochemistry

**Figure 4:**
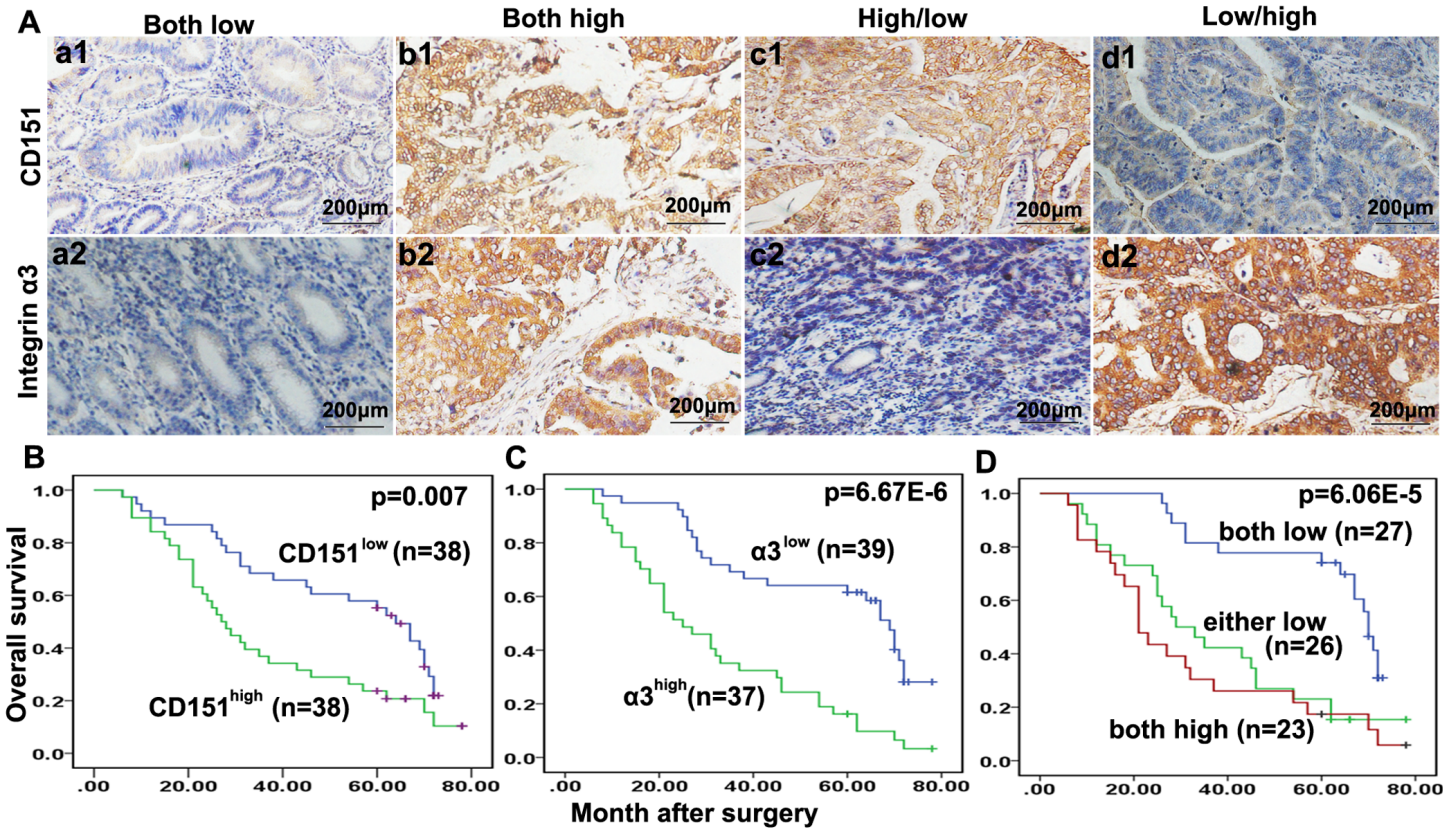
Expression of CD151 or integrin α3 was positively Associated with Malignant Phenotypes of HGC. (**A**) Immunoreactivity of CD151 and integrinα3 protein was located in the cell membranes. In the tumor tissues, CD151 expression showed considerable heterogeneity in the different samples (**Fig.4a** **1, b1, c1 and d1**). Positive staining of Integrinα3 was observed in the membrane of the carcinoma cells (**Fig. 4 a2, b2, c2,** and **d2**). (**B, C and D**) Prognostic significance assessed by Kaplan-Meier survival estimates and log-rank tests. Comparisons of OS by CD151 (B), Integrinα3 (C), and Co-overexpression of CD151 and integrinα3 (D).

The expression of CD151 and integrin α3 protein was investigated in 76 primary HGC patients using tissue microarrays(TMA). CD151 protein immunoreactivity localized to the cell membrane (**Fig. 4A**). In tumor tissues, CD151 expression showed considerable heterogeneity in the different samples (**Figs. 4a** **1, b1, c1 and d1**). CD151^high^ accounted for 50% (38/76) of the whole cohort. As shown in Table 1, CD151^high^ was significantly correlated with tumor size (*p = *0.021), depth of invasion (*p = *0.004), lymph node involvement (*p = *0.028) and high tumor stage (*p = *0.002). However, other clinical characteristics, including age, sex and tumor differentiation, were not significantly related to the expression of CD151.

The membranes of tumor cells stained positive for integrin α3 (**Figs. 4a** **2, b2, c2, and d2**). In all the tissues analyzed, high levels of integrin α3 expression were detected in 27 HGC tissue samples (35.52%). Consistent with the results of previous studies [16], [18], patients with high integrin α3 expression were more likely to exhibit aggressive features. Integrin α3^high^ patients showed larger tumors (*p = *3.46E−4), greater depth of invasion (*p = *0.001), higher tumor stage (*p = *0.005), and more lymph node involvement (*p = *0.040) than patients with low integrin α3 expression (**Table 1**).

### Overexpression of CD151 and/or Integrin α3 are Independent Factors Predicting the Prognosis of HGC Patients

Up to the last follow-up, the 3- and 5-year OS rates in the whole population were 53.0 and 40.78%, The 5-year OS in the CD151^low^ group was significantly higher than that in the CD151^high^ group (68.42% *vs.* 23.68%, respectively, *p* = 0.007, **Fig. 4B**), and the postoperative 5-year OS of HGC patients was higher in the integrin α3^low^ than in the integrin α3^high^ group (66.67% *vs.* 13.51%, *p* = 6.67E−6). Evaluation of the combined effect of CD151 and integrin α3 on the prognosis of HGC showed that the 5-year OS of CD151^high^/integrin α3^high^ patients (group III, n = 23) was 17.40%, which was significantly lower than that of CD151^low^/integrin α3^low^ patients (77.78% group I, n = 27) and either low patients (patients with either low CD151 or low integrin α3 alone)(23.08%, group II, n = 26, **Figs. 4C and D**).

Univariate analysis showed that tumor size, depth of invasion, lymph node involvement, high tumor stage, high CD151 expression, high level of integrin α3 and co-expression of CD151 and integrin α3 were predictors for OS. Other characteristics including age, sex and differentiation had no prognostic significance for OS (**Table** **2**). Multivariate Cox proportional hazards model showed that depth of invasion was an independent prognostic indicator for OS (**Table. 2**).

**Table 2:** Univariate and Multivariate analysis of factors associated with 3-year survival.

Variables	Univariate analysis		Multivariate analysis	
	Hazard ratio (95% CI)	*p*	Hazard ratio (95% CI)	p
Age (years) (≤65 *vs.* >65)	1.007 (0.549–1.709)	0.979	n.a.	
Gender (male vs. female)	0.661(0.387–1.128)	0.129	na	
Tumor size, cm (≤5.5 vs. >5.5)	2.273(1.345–3.840)	**0.002**	0.574(0.237–1.391)	0.219
Differention (I-II vs. III)	0.674 (0.390–1.408)	0.158	n.a.	
Depth of invasion (T1 vs. T2–T4)	2.799 (1.501–5.221)	**0.001**	2.997(1.486–6.043)	**0.002**
Lymph nodule involvement (N0 vs. N1/N2/N3)	2.180(1.228–3.868)	**0.008**	0.590 (0.160–2.177)	0.429
Stage (I vs. II/III/IV)	2.060(1.148–3.698)	**0.015**	1.882(0.536–6.606)	0.324
CD151(low vs. high)	1.990 (1.181–3.353)	**0.010**	0.443(0.118–1.583)	0.206
Integrin α3 (low vs. high)	3.197(1.861–5.432)	**6.84E−5**	1.329(0.421–4.193)	0.627
Co-expression of CD151/Integrinα3	1.869 (1.377–2.536)	**2.32E−5**	3.367(0.934–12.140)	0.064

**Abbreviations and Note**: Cox proportional hazards regression model, 95%CI, 95% confidence interval; Multivariate analysis, Cox proportional hazards regression model. Variables were adopted for their prognostic significance by univariate analysis with forward stepwise selection (Forward, likelihood ratio). Variables were adopted for their prognostic significance by univariate analysis (*p*<0.05).

## Discussion

The results of the present study showed that CD151 was expressed at higher levels in GC cells and tumor tissues than in HGEC cells and nontumor tissues, which is consistent with previous reports on CD151 expression in a variety of tumors, including intrahepatic cholangiocarcinoma, HCC, breast, lung, colon and prostate cancer [15], [19], [20], [21]. Furthermore, our study showed that CD151 forms a functional complex with integrin α3, and downregulation of CD151 or integrin α3 expression markedly inhibited the invasion and metastasis of HGC cells in vitro. Clinically, our results indicated that high level of CD151 expression or the co-overexpression of CD151 and integrin α3 may have unfavorably prognostic implications for patients with GC.

The initial evidence that CD151 promotes metastasis came from a study showing that an antibody with unknown specificity inhibited metastasis formation by a human epidermoid carcinoma line *in vivo*. The antibody recognized CD151 and inhibited cell migration without affecting adhesion or proliferation [22]. Overexpression of CD151 has been detected in many tumor types, and increased CD151 expression has been associated with a poor prognosis in breast, pancreatic, and non-small-cell lung cancers as well as HCC. Furthermore, CD151 has been shown to be a better predictor of prognosis in patients with prostate cancer than histological grading [23]. In the present study, two lines of evidence indicated that overexpression of CD151 is of clinical significance in HGC. First, CD151 overexpression was more frequently observed in HGC patients with poor prognosis. Second, increased expression of CD151 enhanced the invasion and metastasis of HGC cells. In addition, clinical data revealed that elevated CD151 expression outperformed other commonly used clinical parameters such as increased tumor size and poor differentiation for predicting HGC prognosis. Taken together, these findings indicate that CD151 plays an important role in the progression of HGC. In the present study, we show that CD151 interacts with integrin α3 in HGC cells. Modulation of the expression levels of integrin α3 in HGC-27-vshCD151-cDNA-CD151 cells revealed the formation of a functional CD151-integrin α3 complex and showed that co-overexpression of CD151 and integrin α3 was associated with increased metastatic potential of HGC cells. Considering the established role of integrin α3 in HGC [16], it is reasonable to assume that CD151 overexpression may promote metastasis/invasion in GC.

Because of their hydrophobic nature, tetraspanins associate with each other and with other membrane proteins [24]. Indeed, it is well established that tetraspanins provide a signaling platform in the plasma membrane through the formation of tetraspanin-enriched microdomains (TEMs) [6], [7]. To date, different types of membrane proteins, including growth factor receptors, integrins, immunoglobulin domains and EWI-F(a subfamily of Ig proteins) have been found in TEMs [25]. Moreover, the functions of these membrane proteins have been reported to be intimately associated with TEMs. For example, different combinations of tetraspanins forming TEMs were found to affect the function of growth factor receptors and integrin. More importantly, the expression level of individual tetraspanins in TEMs also has a significant effect on the associated proteins [26], [27]. In recent studies, knock-down of CD151 was shown to impair the formation of TEMs and CD151 deletion inhibited the function of several membrane proteins, indicating that CD151 plays a critical role in TEM formation and function [7], [26]. Furthermore, blocking of CD151 markedly impaired the invasiveness and metastatic potential of tumor cells, and targeting the CD151 protein or TEMs has become a promising therapeutic strategy [23]. In the present study, the expression of CD151 was shown to be an independent predictor for OS. However, the combined expression of CD151 and integrin α3 was a more reliable predictor of OS than CD151 or integrin α3 expression alone, supporting the notion that both CD151 and integrin α3 play an important role in HGC. Therefore, our results are significant and suggest that CD151 or the CD151-integrin α3 complex may be important targets in the treatment of patients with GC.

In conclusion, CD151 overexpression is a predictor of poor outcome in patients with HGC, and CD151 or the CD151-integrin α3 complex could be potential targets for the treatment of HGC.

## References

[1] Jemal A, Bray F (2011) Center MM, Ferlay J, Ward E, et al (2011) Global cancer statistics. CA Cancer J Clin 61: 69–90. 21296855 10.3322/caac.20107

[2] Ohtsu A (2008) Chemotherapy for metastatic gastric cancer: past, present, and future. J Gastroenterol 43: 256–264. 18458840 10.1007/s00535-008-2177-6

[3] Zhao ZS, Wang YY, Chu YQ, Ye ZY, Tao HQ (2010) SPARC is associated with gastric cancer progression and poor survival of patients. Clin Cancer Res 16: 260–268. 20028745 10.1158/1078-0432.CCR-09-1247

[4] Wang YY, Ye ZY, Zhao ZS, Tao HQ, Li SG (2010) Systems biology approach to identification of biomarkers for metastatic progression in gastric cancer. J Cancer Res Clin Oncol 136: 135–141. 19649653 10.1007/s00432-009-0644-y PMC11828270

[5] Wu MS, Shun CT, Wang HP, Sheu JC, Lee WJ, et al (1997) Genetic alterations in gastric cancer: relation to histological subtypes, tumor stage, and Helicobacter pylori infection. Gastroenterology 112: 1457–1465. 9136822 10.1016/s0016-5085(97)70071-4

[6] Yanez-Mo M, Barreiro O, Gordon-Alonso M, Sala-Valdes M, Sanchez-Madrid F (2009) Tetraspanin-enriched microdomains: a functional unit in cell plasma membranes. Trends Cell Biol 19: 434–446. 19709882 10.1016/j.tcb.2009.06.004

[7] Hemler ME (2005) Tetraspanin functions and associated microdomains. Nat Rev Mol Cell Biol 6: 801–811. 16314869 10.1038/nrm1736

[8] Wright MD, Moseley GW, van Spriel AB (2004) Tetraspanin microdomains in immune cell signalling and malignant disease. Tissue Antigens 64: 533–542. 15496196 10.1111/j.1399-0039.2004.00321.x

[9] Hemler ME (2008) Targeting of tetraspanin proteins–potential benefits and strategies. Nat Rev Drug Discov 7: 747–758. 18758472 10.1038/nrd2659 PMC4737550

[10] Ashman LK (2002) Cd151. J Biol Regul Homeost Agents 16: 223–226. 12456024

[11] Zijlstra A, Lewis J, Degryse B, Stuhlmann H, Quigley JP (2008) The inhibition of tumor cell intravasation and subsequent metastasis via regulation of in vivo tumor cell motility by the tetraspanin CD151. Cancer Cell 13: 221–234. 18328426 10.1016/j.ccr.2008.01.031 PMC3068919

[12] Lazo PA (2007) Functional implications of tetraspanin proteins in cancer biology. Cancer Sci 98: 1666–1677. 17727684 10.1111/j.1349-7006.2007.00584.x PMC11159418

[13] Shi GM, Ke AW, Zhou J, Wang XY, Xu Y, et al (2010) CD151 modulates expression of matrix metalloproteinase 9 and promotes neoangiogenesis and progression of hepatocellular carcinoma. Hepatology 52: 183–196. 20578262 10.1002/hep.23661

[14] Zheng B, Liang L, Wang C, Huang S, Cao X, et al (2011) MicroRNA-148a suppresses tumor cell invasion and metastasis by downregulating ROCK1 in gastric cancer. Clin Cancer Res 17: 7574–7583. 21994419 10.1158/1078-0432.CCR-11-1714

[15] Ke AW, Shi GM, Zhou J, Wu FZ, Ding ZB, et al (2009) Role of overexpression of CD151 and/or c-Met in predicting prognosis of hepatocellular carcinoma. Hepatology 49: 491–503. 19065669 10.1002/hep.22639

[16] Saito Y, Sekine W, Sano R, Komatsu S, Mizuno H, et al (2010) Potentiation of cell invasion and matrix metalloproteinase production by alpha3beta1 integrin-mediated adhesion of gastric carcinoma cells to laminin-5. Clin Exp Metastasis 27: 197–205. 20352300 10.1007/s10585-010-9314-3

[17] Ke AW, Shi GM, Zhou J, Huang XY, Shi YH, et al.. (2011) CD151 amplifies signaling by integrin alpha6beta1 to PI3K and induces the epithelial-mesenchymal transition in HCC cells. Gastroenterology 140: 1629–1641 e1615. 10.1053/j.gastro.2011.02.008 21320503

[18] Takatsuki H, Komatsu S, Sano R, Takada Y, Tsuji T (2004) Adhesion of gastric carcinoma cells to peritoneum mediated by alpha3beta1 integrin (VLA-3). Cancer Res 64: 6065–6070. 15342388 10.1158/0008-5472.CAN-04-0321

[19] Huang XY, Ke AW, Shi GM, Ding ZB, Devbhandari RP, et al (2010) Overexpression of CD151 as an adverse marker for intrahepatic cholangiocarcinoma patients. Cancer 116: 5440–5451. 20715158 10.1002/cncr.25485

[20] Kwon MJ, Park S, Choi JY, Oh E, Kim YJ, et al (2012) Clinical significance of CD151 overexpression in subtypes of invasive breast cancer. Br J Cancer 106: 923–930. 22294188 10.1038/bjc.2012.11 PMC3306846

[21] Tokuhara T, Hasegawa H, Hattori N, Ishida H, Taki T, et al (2001) Clinical significance of CD151 gene expression in non-small cell lung cancer. Clin Cancer Res 7: 4109–4114. 11751509

[22] Testa JE, Brooks PC, Lin JM, Quigley JP (1999) Eukaryotic expression cloning with an antimetastatic monoclonal antibody identifies a tetraspanin (PETA-3/CD151) as an effector of human tumor cell migration and metastasis. Cancer Res 59: 3812–3820. 10447000

[23] Haeuw JF, Goetsch L, Bailly C, Corvaia N (2011) Tetraspanin CD151 as a target for antibody-based cancer immunotherapy. Biochem Soc Trans 39: 553–558. 21428938 10.1042/BST0390553

[24] Kovalenko OV, Metcalf DG, DeGrado WF, Hemler ME (2005) Structural organization and interactions of transmembrane domains in tetraspanin proteins. BMC Struct Biol 5: 11. 15985154 10.1186/1472-6807-5-11 PMC1190194

[25] Hemler ME (2001) Specific tetraspanin functions. J Cell Biol 155: 1103–1107. 11756464 10.1083/jcb.200108061 PMC2199333

[26] Rubinstein E (2011) The complexity of tetraspanins. Biochem Soc Trans 39: 501–505. 21428928 10.1042/BST0390501

[27] Stipp CS, Kolesnikova TV, Hemler ME (2003) Functional domains in tetraspanin proteins. Trends Biochem Sci 28: 106–112. 12575999 10.1016/S0968-0004(02)00014-2

